# Emergence of Novel Norovirus GII.4 Variant 

**DOI:** 10.3201/eid3001.231003

**Published:** 2024-01

**Authors:** Preeti Chhabra, Damien C. Tully, Janet Mans, Sandra Niendorf, Leslie Barclay, Jennifer L. Cannon, Anna M. Montmayeur, Chao-Yang Pan, Nicola Page, Rachel Williams, Helena Tutill, Sunando Roy, Cristina Celma, Stuart Beard, Michael L. Mallory, Gédéon Prince Manouana, Thirumalaisamy P. Velavan, Ayola Akim Adegnika, Peter G. Kremsner, Lisa C. Lindesmith, Stéphane Hué, Ralph S. Baric, Judith Breuer, Jan Vinjé

**Affiliations:** Centers for Disease Control and Prevention, Atlanta, Georgia, USA (P. Chhabra, L. Barclay, J.L. Cannon, A.M. Montmayeur, J. Vinjé);; London School of Hygiene & Tropical Medicine, London, UK (D.C. Tully, S. Hué);; University of Pretoria, Pretoria, South Africa (J. Mans, N. Page);; Robert Koch Institut, Berlin, Germany (S. Niendorf);; California Department of Public Health, Richmond, California, USA (C.-Y. Pan);; National Institute for Communicable Diseases, Sandringham, South Africa (N. Page);; UCL Great Ormond Street Institute of Child Health, London (R. Williams, H. Tutill, S. Roy, J. Breuer);; UK Health Security Agency, London (C. Celma, S. Beard);; University of North Carolina, Chapel Hill, North Carolina, USA (M.L. Mallory, L.C. Lindesmith, R.S. Baric);; Universitätsklinikum Tübingen, Tübingen, Germany (G.P. Manouana, T.P. Velavan, A.A. Adegnika);; Centre de Recherches Médicales de Lambaréné, Lambarene, Gabon (G.P. Manouana, A.A. Adegnika, P.G. Kremsner);; Vietnamese-German Center for Medical Research, Hanoi, Vietnam (T.P. Velavan);; Duy Tan University, Da Nang, Vietnam (T.P. Velavan);; German Center for Infection Research, Tübingen (A.A. Adegnika)

**Keywords:** norovirus, viruses, enteric infections, acute gastroenteritis, United States, United Kingdom, South Africa

## Abstract

We detected a novel GII.4 variant with an amino acid insertion at the start of epitope A in viral protein 1 of noroviruses from the United States, Gabon, South Africa, and the United Kingdom collected during 2017–2022. Early identification of GII.4 variants is crucial for assessing pandemic potential and informing vaccine development.

Norovirus is the most common cause of acute gastroenteritis (AGE) worldwide. Norovirus has an ≈7.7 kb positive-sense single-stranded RNA genome organized into 3 open reading frames (ORFs). ORF1 encodes a polyprotein that is posttranslationally cleaved into 6 nonstructural (NS) proteins, including NS7, the viral RNA-dependent RNA polymerase (RdRp). ORF2 encodes the major viral protein (VP), VP1, and ORF3 encodes the minor VP2 capsid protein. 

Noroviruses are genetically diverse and classified into >10 different genogroups. Genogroup II genotype 4 (GII.4) viruses cause most illnesses worldwide (*1*,*2*). GII.4 variants include US95–96, Farmington Hills_2002, Asia_2003, Hunter_2004, Yerseke_2006, Den Haag_2006, Osaka_2007, Apeldoorn_2007, New Orleans_2009, Sydney_2012, and HongKong_2019 (*3*). GII.4 variant emergence has been associated with changes in epitopes A–I on the surface exposed P2 subdomain of VP1 affecting interactions with histo-blood group antigens (HBGA) on host cells (*4*). Since 2012, GII.4 Sydney has been the most prevalent norovirus genotype globally (*2*). We sequenced complete genomes or VP1 of GII.4 viruses from recent outbreaks and sporadic cases that could not be genotyped to investigate genomic similarities with existing variants.

## The Study

Several surveillance networks track trends in norovirus strain diversity, including CaliciNet in the United States (*5*) and NOROPATROL in the United Kingdom. In July 2017, four identical sequences from a norovirus outbreak in a childcare facility in San Francisco, California, USA, were uploaded to CaliciNet (https://www.cdc.gov/norovirus/reporting/calicinet). We genotyped those 4 sequences as GII.4 untypeable; the sequences had >2% nucleotide sequence difference in the 5′ end of ORF2 from existing GII.4 viruses. We identified genetically similar strains in stool specimens from 3 UK outbreaks: 1 strain from a hospitalized 32-year-old patient with AGE in Newcastle in 2019, 1 from a 1-year-old child in London in 2021, and 2 from Brighton in 2021, from a 1-year-old and a 3-year-old from the same household in an oyster-related outbreak. We also identified similar strains in sporadic samples from children with AGE in Gabon during 2018–2019 (*6*) and in children and adolescents with AGE from Western Cape and Gauteng in South Africa during 2021–2022. We sequenced 5 near-complete genomes and 10 complete VP1 sequences and compared those with existing GII.4 sequences from the Human Calicivirus Typing Tool (https://calicivirustypingtool.cdc.gov/gebali.cgi).

We extracted viral RNA and obtained complete genome or VP1 sequences for strains from the United States, United Kingdom, and South Africa according to published methods (*5*–*9*). We amplified the complete VP1 from Gabon strains by seminested reverse transcription PCR (RT-PCR) using Oligo dT and Lunascript Master Mix Kit (New England Biolabs, https://www.neb.com) for cDNA synthesis at 55°C for 30 min. We amplified complete VP1 and VP2 by using seminested RT-PCR and oligonucleotide primers designed for this study (Table). We performed RT-PCRs by using OneTaq 2X Master Mix (New England Biolabs) for 30 cycles at 94°C for 10 s, 45°C for 30 s, and 72°C for 3 min, then a final extension of 72°C for 2 min. We sequenced all amplicons. 

**Table:** List of oligonucleotide primers used to examine emergence of novel norovirus GII.4 strains on 3 continents*

Primer names	Sequence, 5′ → 3′	Orientation	Nested reverse transcription PCR
NV6e	ACC AYT WTG ATG CAG ACT A	Forward	Round 1
NV6f	ACC AYT ATG ATG CTG ATT A	Forward	Round 1
NV6g	ATC AYT ATG ATG CWG AYT A	Forward	Round 1
NV357a	CGC CAG TCC AGG AGT CCA AAA TY	Reverse	Round 1
NV378	GCT TAC GAA TGT GAG CGA A	Reverse	Round 2

*Primers were used to amplify complete ORF2 and ORF3 genes of GII.4 San Francisco strains from Gabon. ORF, open reading frame

We aligned complete VP1 amino acid sequences with GII.4 reference strains representing all known emerging and epidemic GII.4 viruses by using ClustalW in MEGA X (*10*). We computed maximum-likelihood phylogenetic trees by using the Jones-Taylor-Thornton model for amino acid sequences and Tamura-Nei model for nucleotide sequences and performed gamma distribution of evolutionary rates among sites using 100 bootstrap replications. We deposited nucleotide sequences of GII.4 San Francisco strains in GenBank (accession nos. OR262322–29, OR262341–44, and MW506847–49). We predicted 3-dimensional structures of GII.4 San Francisco viruses by using ChimeraX version 1.4 (*11*) and the alphafold prediction tool (*12*) and used the P-domain of GII.4 Sydney (Protein Data Bank no. PDB 4OP7; https://www.rcsb.org/structure/4OP7) as the backbone. To evaluate the effects of amino acid changes in P2, we synthesized virus-like particles from the codon-optimized ORF2 sequence of SF128 (GenBank accession no. OR262322) and compared ligand binding with other GII.4 virus-like particles (*4*).

**Figure 1:**
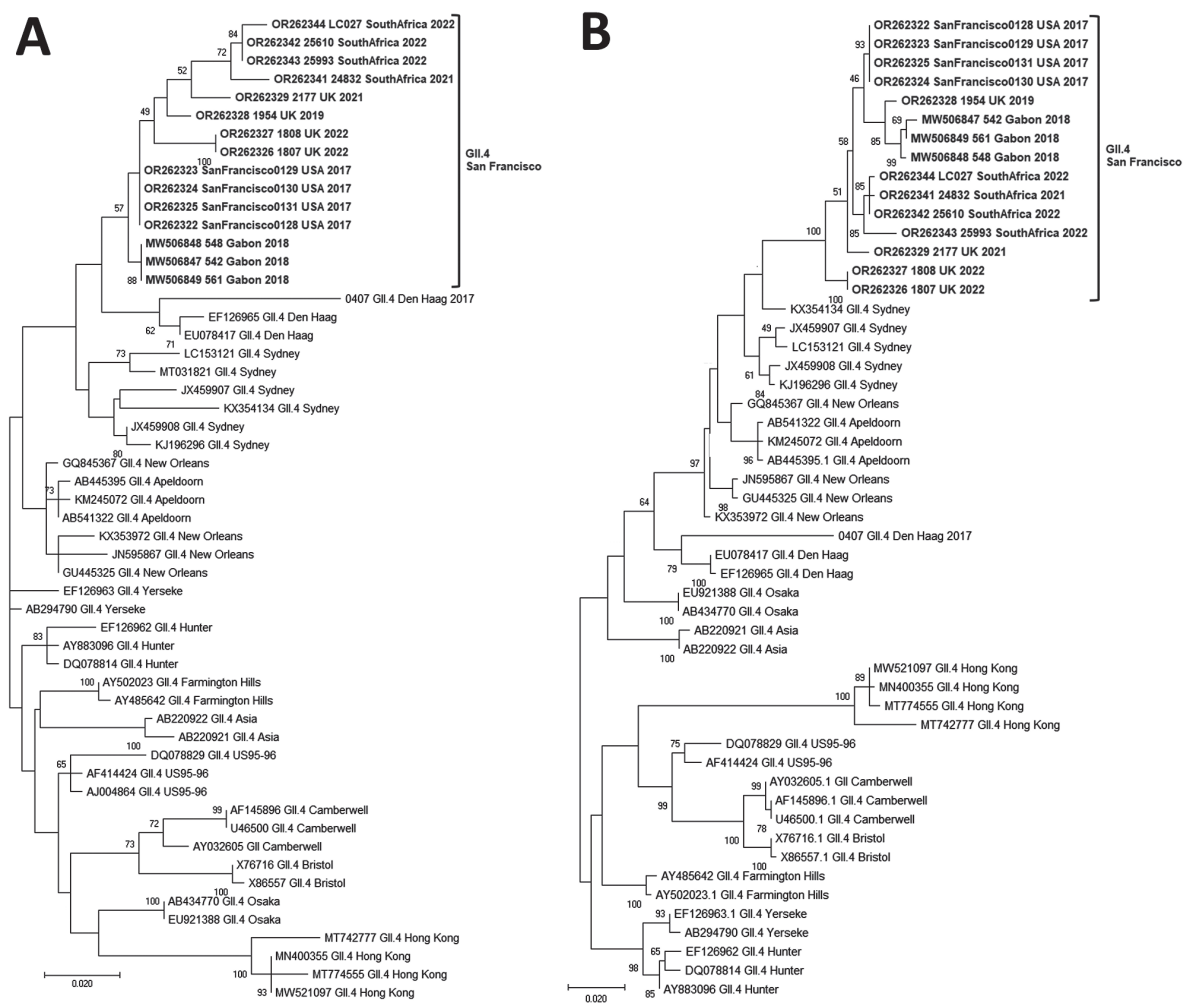
Phylogenetic trees of the emerging novel norovirus GII.4 strains on 3 continents. A) Common genotyping region C; 250 nt from the 5′ end of ORF2; B) complete VP1 aa sequences. Phylogenetic trees show novel GII.4 San Francisco strains and GII.4 variants, including recently identified clusters like GII.4 Hong Kong. Evolutionary analyses were conducted in MEGA X (https://www.megasoftware.net) using the maximum-likelihood method based on the Tamura-Nei model for the C region and Jones-Taylor-Thornton matrix-based model for VP1. We used a discrete gamma distribution to model evolutionary rate differences among sites; 5 categories γ parameter = 0.2174. Bootstrap (100) values are indicated at the nodes. Trees were drawn to scale. Scale bar represents nucleotide substitutions per site. ORF, open reading frame; VP1, viral protein 1.

We found that GII.4 San Francisco sequences from the 5′-end of ORF2 were closest to GII.4 Sydney and GII.4 Den Haag reference strains with maximum identities ranging from 91%–95% (Figure 1, panel A). Complete VP1 amino acid sequences of GII.4 San Francisco strains formed a distinct cluster with 5%–10% amino acid difference from GII.4 New Orleans and GII.4 Sydney (Figure 1, panel B). We typed RdRp sequences of all strains as GII.P31.

Of note, VP1 sequences of all GII.4 San Francisco strains had an alanine insertion at position 293/294 at the start of epitope A, coinciding with a unique SVTQTAT/A motif at positions 289–295 adjacent to epitope A (Appendix Figure 1). Compared with GII.4 Sydney_2012 and GII.4 New Orleans viruses, we observed mutations at amino acid residues 256 and 438 in the P1 region and 294, 310, 340, 341, 356, 372, 373, 377, 393, and 395 in the hypervariable region, P2 (Appendix Figure 1).

**Figure 2:**
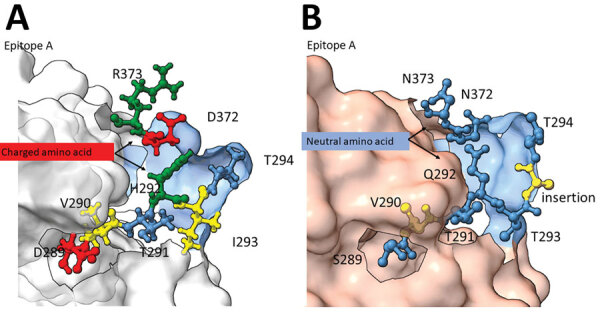
Structural changes of emergent novel norovirus GII.4 strains from 3 continents. A) Sydney GII.4 strain (GenBank accession no. JX459908); B) GII.4 San Francisco strain. The 3-dimensional structure models were predicted by using ChimeraX version 1.4 (*11*) and the alphafold prediction tool (*12*). Models show structural changes near and within the epitope A antigenic region on GII.4 San Francisco P-domain (panel B) are overlayed on a GII.4 Sydney 2012 backbone (Protein Data Bank, https://www.rcsb.org/structure/4OP7). Negatively (red) and positively (green) charged amino acids of GII.4 Sydney (panel A) were replaced with neutral amino acids (blue) in the GII.4 San Francisco strain and a hydrophobic (yellow) amino acid, alanine, was inserted between T293 and T294.

Homology modeling of the GII.4 San Francisco P-domain using GII.4 Sydney 2012 as a backbone (PDB 4OP7; GenBank accession no. JX459908) showed structural changes near and within epitope A (Figure 2). When the alanine insertion and SVTQTAT/A motif were introduced, several charged amino acids in GII.4 Sydney_2012 were replaced by neutral amino acids (Figure 2). We also observed changes in the charge or hydrophobicity of amino acids in the monoclonal antibody binding epitope G (A356N) and within and around the HBGA binding regions D391N, S393D, and T395A, except in strains from South Africa (Appendix Figure 1). The alanine insertion in GII.4 San Francisco strains does not ablate binding to ligands found in porcine gastric mucin (Appendix Figure 2), which is consistent with ligand binding patterns known to correlate with susceptibility.

## Conclusions

We report a novel norovirus GII.4 variant, named GII.4 San Francisco, detected in human stool specimens from patients with AGE on at least 3 continents during 2017–2022. The novel strains have a unique amino acid insertion in VP1 at the start of epitope A. We observed a similar unique insertion on epitope D in GII.4 variant Farmington Hills, which emerged in 2002, replacing the GII.4 US95–96 viruses, which had been circulating globally since 1995 (*13*). Whether the emerging GII.4 San Francisco strains will replace the current globally dominant GII.4 Sydney variant is not yet clear. Previous studies showed that epidemic GII.4 viruses diversified and spread over wide geographic areas for several years before epidemic emergence (*14*).

GII.4 viruses have always had strong immunodominance on epitope A, and alterations in epitope A residues has affected antibody responses (*15*). Addition of alanine at the start of epitope A and introduction of several neutral amino acids (SVTQTAT/A) before the insertion indicate major changes in the structure that could have an outsize effect on neutralizing antibody responses. GII.4 San Francisco strains showed mutations at residues S393D and T395A in epitope D. Those changes kept the ligand binding stabilizing function; epitope D also is a neutralizing epitope and an HBGA binding site (*4*). That finding further indicates that this virus has potential for increased spread and warrants additional antigenicity studies. Those data provide information for evaluation of norovirus vaccines that are currently in clinical trials.

In conclusion, but the unique amino acid insertion in epitope A of VP1 together with a >5% aa difference from existing GII.4 variants confirmed that GII.4 San Francisco can be classified as a new GII.4 variant. This virus variant is circulating on at least 3 continents, North America, Europe, and Africa. Early detection and rapid assigning of an agreed upon name for future GII.4 variants will be crucial to assessing their pandemic potential. 

Appendix Additional information on emergent novel norovirus GII.4 strains on 3 continents.
