# Supplementary material for: Emergence of Novel Norovirus GII.4 Variant
Source: Emerg Infect Dis. 2024 Jan;30(1):163–7. doi: 10.3201/eid3001.231003 (PMC10756382; doi:10.3201/eid3001.231003)
Supplement: Appendix — Additional information on emergent novel norovirus GII.4 strains on 3 continents. [file 23-1003-Techapp-s1.pdf]

Article DOI: https://doi.org/10.3201/eid3001.231003

EID cannot ensure accessibility for supplementary materials supplied by authors. Readers who have difficulty accessing supplementary content should contact the authors for assistance.

Emergence of Novel Norovirus GII.4 Variant

Appendix

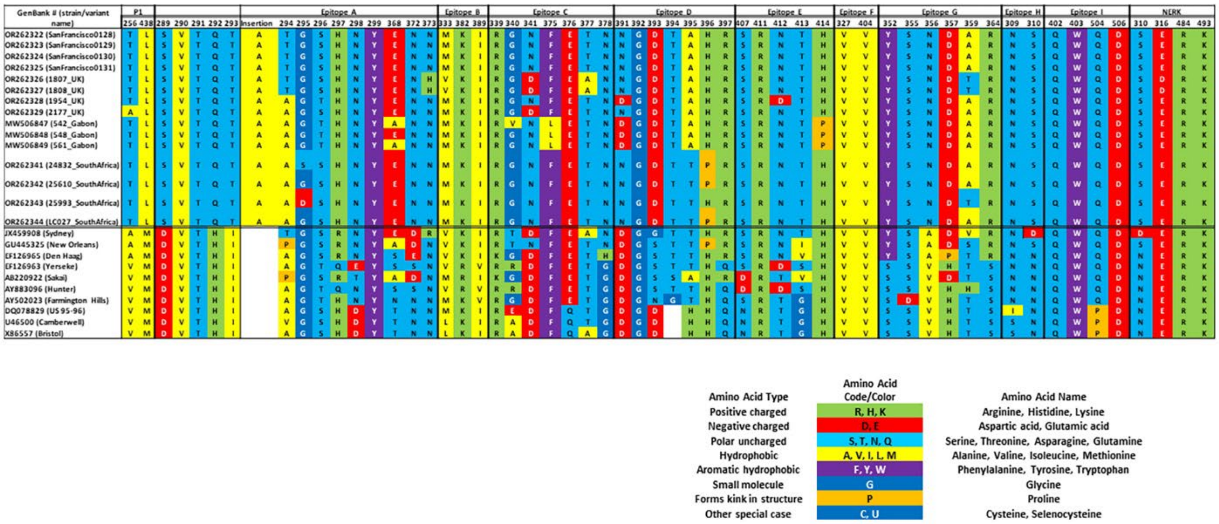

Appendix Figure 1. Amino acid differences of emergent novel norovirus GII.4 strains on 3 continents. Differences in the P1 and P2 antibody binding epitopes of the GII.4 San Francisco strains were compared with reference strains of other epidemic GII.4 variants.

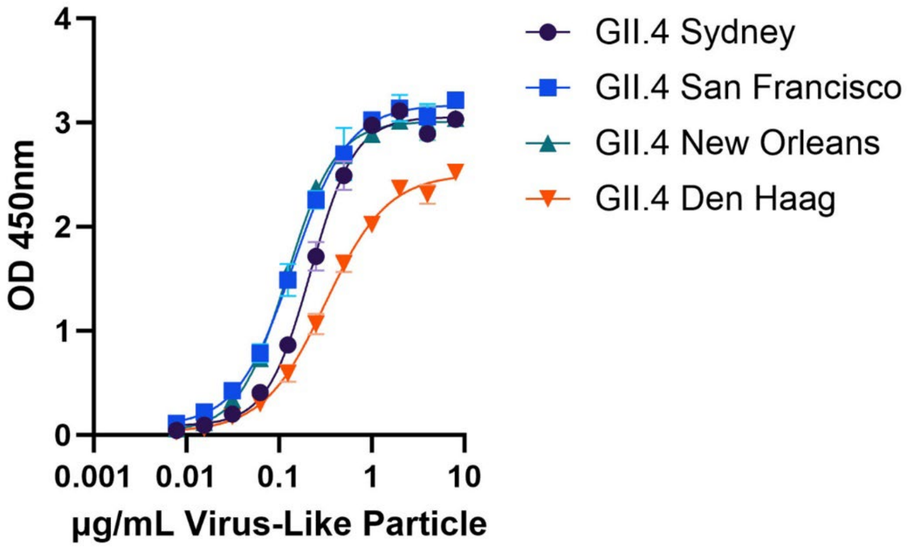

Appendix Figure 2. Optical density of emergent novel norovirus GII.4 strains from 3 continents. GII.4 variants bind to ligands (porcine gastric mucin). The binding of each GII.4 variant was determined across a series of concentrations beginning at 8 µg/mL and the optical density measurements at 450 nm and fit using nonlinear regression analysis (log(agonist) versus response-variable slope) in GraphPad Prism 9.5.1 (GraphPad Software Inc., https://www.graphpad.com). Each virus-like protein was tested in duplicate in 2 independent experiments and mean +/− SEM of all data plotted.
